# Association between persistent smoking after a diagnosis of heart failure and adverse health outcomes: A systematic review and meta-analysis

**DOI:** 10.18332/tid/116411

**Published:** 2020-01-20

**Authors:** Youn-Jung Son, Hyeon-Ju Lee

**Affiliations:** 1 Red Cross College of Nursing, Chung-Ang University, Seoul, Republic of Korea; 2 Department of Nursing, Tongmyong University, Busan, Republic of Korea

**Keywords:** heart failure, smoking, mortality, readmission

## Abstract

**INTRODUCTION:**

Heart failure (HF) is associated with increased mortality worldwide. Adverse health outcomes in HF are commonly attributed to poor adherence to self-care, including smoking cessation. Smoking is the major modifiable risk factor for HF. Patients have been observed to continue smoking even after diagnosis with HF. Despite the possible association between persistent smoking and adverse health outcomes among HF populations, no consensus has been reached. We aimed to review the literature to determine the association between smoking status after HF diagnosis and adverse health outcomes.

**METHODS:**

A systematic literature search was performed in PubMed, PsycINFO, Web of Science, and Embase. Hand searching was also performed. In total, 9 articles (n=70461) were included in the review for meta-analysis, including seven cohort studies and two cross-sectional studies. Quality was assessed using the modified version of the Newcastle–Ottawa Scale.

**RESULTS:**

Approximately 16% of HF patients continued smoking after HF diagnosis. Persistent smoking increased the hazard ratio (HR) of mortality by 38.4% (HR=1.384; 95% CI: 1.139–1.681) and readmission by 44.8% (HR=1.448; 95% CI: 1.086–1.930). Our review also found that persistent smoking was associated with poor health status, ventricular tachycardia, and arterial stiffness.

**CONCLUSIONS:**

This review highlights the importance of assessment for any history of smoking before and after HF diagnosis. There is a need for smoking cessation programs to be established as crucial components of care for patients with HF. More studies are needed to investigate the possible mechanisms underlying relations among smoking patterns and health consequences.

## INTRODUCTION

Heart failure (HF) is a rapidly growing public health issue affecting at least 2–4% of the global population according to the World Health Organization (WHO)^1^. It is characterized by poor quality of life, recurrent hospitalization, and increased mortality risk, due to worsening HF symptoms^2^. The main goal of HF treatment is to reduce symptoms, morbidity and mortality, and improve the quality of life^3,4^. According to the European Society of Cardiology Guidelines for the diagnosis and treatment of HF^5^, self-management is integral to achieving best patient outcomes, to reduce mortality and improve quality of life. Such self-care management includes smoking cessation, blood pressure control, lipid management, weight reduction, and symptom monitoring^6^.

Several epidemiological studies have documented an association between lifestyle variables, such as smoking, obesity and dietary pattern, and HF risk in the general population^6-8^. In particular, smoking is the most significant modifiable risk factor for cardiovascular disease (CVD) including HF^9^. The WHO reported that 10% of all deaths due to CVD can be attributed to smoking^1^. Importantly, persistent smoking after HF diagnosis has been shown to worsen the long-term outcomes in HF and to reduce the efficacy of HF treatment^10^. Specifically, smoking not only increases the risk of coronary artery disease, a major cause of HF, but also increases systolic and diastolic blood pressure and oxidative stress. Furthermore, smoking can lead to vascular inflammation, worsening endothelial function and renal function, all of which have been implicated in the pathophysiology of HF^11^.

Previous studies have reported that despite the recommendation to quit smoking after CVD diagnosis, many patients continued smoking, and their smoking behaviors varied extensively^10,12^. A study by Lim et al.^13^ reported that approximately 50% of smokers continued smoking even after CVD events. Another study also reported that about 16% of participants with HF were currently smoking^14^. A meta-analysis of 14 studies showed that the smoking cessation rates after ischemic heart disease ranged from 7% to 63%^15^. Some studies have suggested that poor adherence to HF treatment, including smoking cessation, increases the risk of adverse health outcomes^15,16^.

Despite this possible association between persistent smoking and adverse health outcomes among HF populations, to our knowledge, there has been no consensus on the magnitude of smoking-related morbidity and mortality in patients with HF^12,13^. A recent review reported that more cardiovascular events in heavier smokers have failed to find a significant negative correlation between smoking habits and cardiovascular risk^10^. Furthermore, few studies have systematically reviewed the literature regarding this relationship. Therefore, the purpose of this study was to examine the association between persistent smoking and the risk of adverse health outcomes in patients with HF, through a critical appraisal of the literature.

## METHODS

### Search strategies

The current study was performed according to The Meta-analysis of Observational Studies in Epidemiology (MOOSE) guidelines^17^. We used the Patient, Interest (or Intervention), Comparison, and Outcomes (PICO) strategy for the construction of the research question of this review: ‘Does persistent smoking (Interest) influence adverse health outcomes (Outcomes) among HF patients (Patients)?’. The ‘C’ element in PICO was not used since the objective of this review did not include clinical trials. The search strategy used was: [smoking OR tobacco OR nicotine OR cigarette smoking) AND (heart failure OR cardiac failure OR heart decompensation OR congestive heart failure OR chronic heart failure) AND (risk OR adverse outcome OR morbidity OR mortality OR readmission OR emergency room visit OR heart rate variability OR heart attack OR ischemic heart disease)].

To identify all relevant articles, published up to 30 June 2019, we conducted an extensive electronic literature search in PubMed (from 1946), PsycINFO (from 1806), Web of Science (from 1900), and Embase (from 1947). Gray literature was searched for additional potentially relevant articles. Hand search was also performed.

### Study selection

**Figure 1:**
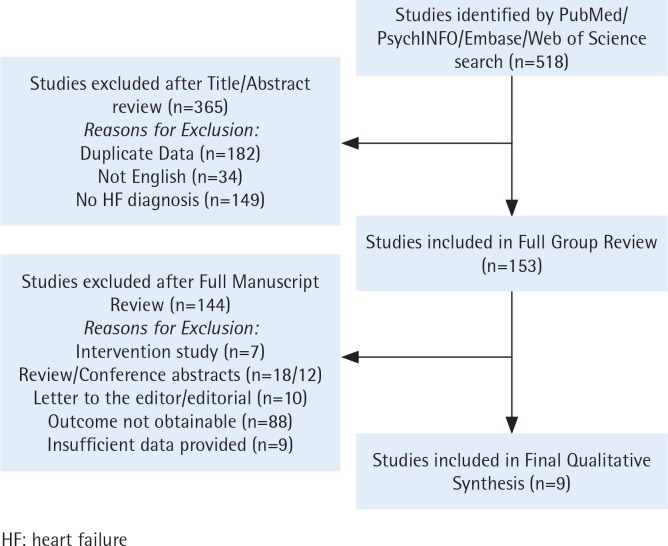
PRISMA flow diagram for study selection

Two independent reviewers screened the titles and abstracts of each included study. At this stage, the reviewers extracted possibly all relevant studies. To be included, studies and peer-reviewed articles needed to be: 1) published in English, and 2) original research using any observational study design (e.g. cross-sectional or cohort) that reported on smoking status in patients with HF as a primary study group. Studies excluded were: 1) intervention studies, reviews, conference abstracts, and letters; 2) studies with no outcome; and 3) studies with insufficient data. Disagreements on eligibility were reconciled by the reviewers. Because no primary data were to be collected, approval from an ethics committee was not required (Figure 1).

### Outcome measures

Outcome measures included mortality, readmission, length of stay, and incidence of adverse health outcomes; specifically, pathological changes such as artery calcification or self-reported health status (Table 1).

**Table 1:** Characteristics of studies included

*Authors (publication year) / location*	*Study design*	*Follow-Up period (months)*	*Sample size (n)*	*Persistent smokers (%)*	*Smoking assessment*	*Smoking status identified*
Evangelista et al. (2000)/ USA^20^	Retrospective	24	753	336 (44.6)	Medical records	Never/former/current smokers
Suskin et al. (2001)/USA, Canada, Belgium^21^	Prospective	41	6704	1562 (23.3)	NA	Never/former/current smokers
Fonarow et al. (2008)/USA^22^	Prospective	22	48612	7743 (15.9)	Medical records	Never/current smokers
Conard et al. (2009)/USA^14^	Cross-sectional	12	537	84 (15.6)	Self-reported	Never/ former/current smokers
Javaheri et al. (2012)/USA^23^	Prospective	NA	87	15 (17.2)	NA	Non or former/current smokers
Graham et al. (2014)/USA^24^	Retrospective	NA	2043	352 (17.2)	Self-reported	Never/ former/current smokers
Li et al. (2017)/China^25^	Cross-sectional	26	354	39 (11.1)	Medical records	Non or former/current smokers
Eriksson et al. (2018)/ Sweden^26^	Prospective	36	9654	1005 (10.4)	Medical records	Never/ former/current smokers
Sandesara et al. (2018)/ USA^27^	Retrospective	33	1717	116 (6.8)	Self-reported	Never/ former/current smokers

**Table:** 

*Authors (publication year) / location*	*Outcome*	*Adjustments for covariates*	*Main result*
Evangelista et al. (2000)/ USA^20^	Readmissions	Age, gender, race, marital status, alcohol consumption, HF etiology, NYHA class	Current smoking was an independent predictor of readmissions (OR=1.82; 95% CI: 1.17–2.82)
Suskin et al. (2001)/USA, Canada, Belgium^21^	Mortality, readmissions	Age, gender, weight, HR, BP, DM, EF, MI, revascularization, NYHA class, CT ratio	Current smoking increased all-cause mortality (RR=1.31; 95% CI: 1.05–1.63), and readmissions (RR=1.21; 95% CI: 1.07–1.38)
Fonarow et al. (2008)/USA^22^	Length of stay, mortality	Age, gender, race, heart rate, BP, HTN, DM, MI, arrhythmias, LVSD, BNP, COPD, creatinine, hemoglobin, troponin I, sodium	Current smoking decreased length of stay (OR=0.97; 95% CI: 0.94–0.99) and was associated with mortality
Conard et al. (2009)/USA^14^	Disease-specific health status, mortality	Age, gender, race, marital status, BMI, BP, heart rate, NYHA class, MI, EF, ICD, pacemaker, PCI, CABG	Current smoking affected health status (p=0.02) Smoking status was not associated with mortality (OR=1.20; 95% CI: 0.70–2.02)
Javaheri et al. (2012)/USA^23^	VT	Age, ArI, H+	Current smoking was independently associated with the presence of VT (OR=9.96; 95% CI: 1.93–51.48)
Graham et al. (2014)/USA^24^	Cognitive function	Age, gender, education, alcohol consumption, atrial fibrillation, HTN, DM, ischemic cardiomyopathy, BMI, BP, creatinine, hemoglobin	Ever smoking was independently associated with the cognitive function (current smoking β=0.321, p=0.016; former smoking β=0.204, p=0.041)
Li et al. (2017)/China^25^	Arterial stiffness	Age, gender, BMI, LVEF, E/A ratio, FEV1	Current smoking was an independent determinant of arterial stiffness (β=0.121, p=0.013)
Eriksson et al. (2018)/ Sweden^26^	Mortality	Age, heart rate, DM, COPD	Ever smoking was independently associated with all-cause mortality
Sandesara et al. (2018)/ USA^27^	Readmissions, all-cause death	Age, gender, race, BMI, BP, DM, NYHA class, COPD, medication, CHD, stroke, creatinine	Current smoking was independently associated with readmission (HR=1.68; 95% CI: 1.08–2.61), death (HR=1.82; 95% CI: 1.19–2.78), and cardiovascular death (HR=1.85; 95% CI: 1.09–3.14)

### Data extraction

Items extracted as characteristics of the included studies comprised: first author’s surname, publication year, study location, study design, follow-up period, number of study participants, number of persistent smokers, smoking assessment, smoking status identified, outcomes, adjusted variables, and main findings (Table 1).

This study also presented the characteristics of the HF patients included in this review. Patient characteristics included: sex, mean age, mean HF duration, HF identified, mean left ventricular ejection fraction (LVEF), mean B-type natriuretic peptide (BNP), and New York Heart Association (NYHA) functional class. In case of disagreement between the two reviewers, consensus was reached after discussion (Table 2).

**Table 2:** Characteristics of HF patients in the included studies

*Authors (publication year)*	*Sex (%)*	*Mean age or range (years)*	*Mean HF duration (years)*	*HF identified*	*Mean LVEF or range (%)*	*Mean BNP or range (pg/mL)*	*NYHA functional class (%)*
Evangelista et al. (2000)^20^	M: 98.8 F: 1.2	69 (33–99)	5.73	Medical records (ICD-9 codes)	NA	NA	III/IV = 13.9/0.8
Suskin et al. (2001)^21^	M: 84.6 F: 15.4	59.4	NA	Medical records (LVEF, NYHA)	25.3	NA	III/IV = 13.9/0.8
Fonarow et al. (2008)^22^	M: 48.0 F: 52.0	73.1	NA	Medical records (LVEF)	39	827.3	NA
Conard et al. (2009)^14^	M: 76.2 F: 23.8	NA	NA	Medical records (LVEF, NYHA class, BNP)	<40	359.4	III/IV = 41.3/5.7
Javaheri et al. (2012)^23^	Male only	64	NA	Medical records (LVEF, NYHA class)	25	NA	III = 15.5
Graham et al. (2014)^24^	M: 80.0 F: 20.0	60.8	NA	Medical records (LVEF, NYHA class)	≤35	NA	III/IV = 29.2/1.2
Li et al. (2017)^25^	M: 47.5 F: 52.5	68.2	NA	Medical records (LVEF)	55.3	138 (96–152)	NA
Eriksson et al. (2018)^26^	M: 53.8 F: 46.2	77.3	>6 months	Medical records (LVEF, NYHA class)	≥40	NA	III/IV = 29.4/1.4
Sandesara et al. (2018)^27^	M: 50.0 F: 50.0	71	NA	Medical records (NYHA class)	NA	NA	III & IV = 35.4

HF: heart failure, LVEF: left ventricular ejection fraction, BNP: B-type natriuretic peptide, NYHA: New York Heart Association, ICD: international classification of diseases, NA: not available.

### Assessment of methodological quality

The Newcastle–Ottawa scale (NOS) for cross-sectional and cohort studies^18^ was used to assess the quality of all included studies. The NOS system consists of 8 items with three subscales: selection of studies, comparability of studies, and the ascertainment of the exposure/outcome of interest. Each item on the scale scored one point. Comparability scored up to two points by adjusting for the topic of interest. Specifically, the maximum score for each study was nine points, with studies scores from five to nine considered of satisfactory quality. All studies were independently rated by two reviewers to assess the quality. Any disagreements in quality assessment were resolved via discussion until consensus was reached.

Table 3 shows the scores regarding the validity of included studies. We considered that the studies with NOS scores of five or greater indicated moderate to high quality studies^18^.

**Table 3:** Quality assessment of the included studies

*Study*	*Newcastle–Ottawa Scale*			
*Authors (publication year)*	*Selection*	*Comparability*	*Outcome*	*Total score*
Evangelista et al. (2000)^20^	4	1	2	7
Suskin et al. (2001)^21^	4	1	3	8
Fonarow et al. (2008)^22^	4	1	2	7
Conard et al. (2009)^14^	2	2	3	7
Javaheri et al. (2012)^23^	2	1	2	5
Graham et al. (2014)^24^	3	1	2	6
Li et al. (2017)^25^	4	2	3	9
Eriksson et al. (2018)^26^	3	1	3	7
Sandesara et al. (2018)^27^	3	1	2	6

### Data synthesis

We investigated how smoking status affected health in patients diagnosed with HF. These estimates were combined using meta-analysis with a random-effects model to derive summary hazard ratio (HR) estimates by outcome.

We used a random-effects model because of the presumed heterogeneity between the studies^19^ . Heterogeneity was analyzed using I^2^ with its 95% confidence interval (CI) and Q statistics (statistical significance was set at p<0.05). The Comprehensive Meta-Analysis software (version 3.0; Biostat, Englewood, NJ, USA) was used to calculate the pooled estimates and forest plots.

## RESULTS

### Overview of the included studies

We initially confirmed a total of 518 studies that met our search criteria. After performing a title and abstract review, 365 studies were excluded resulting in 153 studies that underwent full-text review. Finally, nine studies (five cohort^20-24^, two cross-sectional^14,25^, and two recent cohort^26,27^) were included in the review (Figure 1).

Table 1 summarizes the articles and their findings. The follow-up period ranged from 12 to 41 months and the sample size ranged from 87 to 48612 with a total of 70461, of which, 11252 (16%) continued to smoke after HF diagnosis. Smoking status was categorized as current smokers and non-smokers including former or never smokers. Information on smoking history was obtained via self-reported questionnaires and medical records. Using Newcastle–Ottawa’s quality tools, most of the studies were considered to be of good quality, while one study was evaluated to have fair quality. The scores ranged from 5 to 9 (Table 3).

### Characteristics of HF patients included in this review

The demographic data of the HF patients in the studies are given in Table 2. Fifty-four per cent of the samples were men. The mean age range of the study participants was 59.4 to 77.3 years. The duration of HF was reported in two studies as 5.73 years20 and more than 6 months26, respectively. The severity of HF was verified by LVEF and BNP values, and NYHA functional class. In six studies, the LVEF was <40%. Mean BNP was reported in three studies and ranged from 138.0 to 827.3. The NYHA functional classification was reported in seven studies.

### The impact of smoking on adverse health outcomes

In the present study, persistent smoking showed association with mortality (n=5), readmission (n=3), and disease-specific health status (n=1), ventricular tachycardia (n=1), arterial stiffness (n=1), cognitive function (n=1), and length of stay (n=1) after the diagnosis of HF.

#### Mortality

Five studies provided data on mortality among those who continued smoking after HF diagnosis. Three of the studies used prospective cohort study design to assess mortality^21,22,26^, one used a retrospective cohort study design27, while the other used a cross-sectional design^14^. One was conducted in Sweden, while four reported data from the same population in the USA.

**Figure 2:**
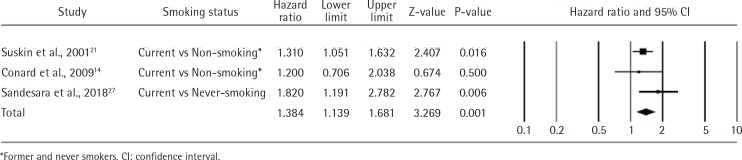
Mortality in current smokers vs non-smokers or never smokers

Three of the included studies were metaanalyses14,21,27. The risk of mortality was greater in current smokers than in non-smokers (combined HR=1.384; 95% CI: 1.139–1.681; n=8958). The pooled results showed low heterogeneity (I2=5.5%; p=0.347) (Figure 2).

Subgroup analysis revealed a higher mortality risk ratio of never smokers versus current smokers of 1.820 (95% CI: 1.191–2.782; 1 study, n=1717) than that of non-smokers versus current smokers (1.402; 95% CI: 0.955–2.060; 2 studies, n=7241). Although two studies noted that continuous smoking after HF diagnosis was independently associated with mortality, they provided no statistical data^22,26^.

#### Readmission rates

**Figure 3:**
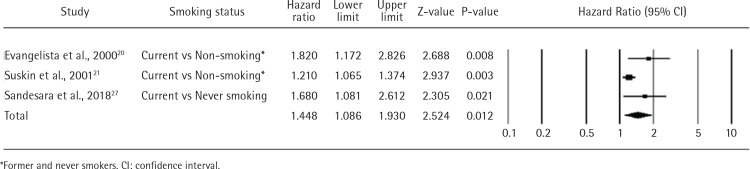
Readmissions for heart failure in current smokers vs non-smokers or never smokers

Three studies reported on the association between readmission and persistent smoking after HF diagnosis. Two of such studies used retrospective cohort study design to assess readmission^20,27^ while the other was a prospective cohort study21. All studies were conducted in the US. The risk of readmission was greater in current smokers than in non-smokers (combined HR=1.448; 95% CI: 1.086–1.930; n=7457). The pooled results showed moderate heterogeneity (I^2^=57.14%; p=0.097) (Figure 3). Subgroup analysis revealed a higher readmission risk ratio of never smokers versus current smokers of 1.680 (95% CI: 1.081–2.612; one study, n=1717) than non-smokers versus current smokers (1.402; 95% CI: 0.955–2.060; 2 studies, n=7457).

#### Other adverse health outcomes

One cross-sectional study conducted in the US examined the disease-specific health status depending on the smoking status after HF diagnosis^14^. Statistically significant differences in the diseasespecific health status were observed between the smoker and non-smoker groups in terms of physical function, symptoms, social function, self-efficacy, and quality of life (F=3.47; p=0.03; n=537). In particular, the disease-specific health status was lower in current smokers than in never smokers (p=0.01) or former smokers (p=0.02). One prospective cohort study conducted in the US examined the incidence of ventricular tachycardia (VT) during sleep depending on the smoking status after HF diagnosis^23^. The incidence of VT was greater in current smokers than in non-smokers (OR=9.96; 95% CI: 1.93–51.48; n=87). One cross-sectional study conducted in China examined the incidence of arterial stiffness depending on the smoking status after HF diagnosis^25^. Current smoking was an independent determinant of arterial stiffness (β=0.121; p=0.013; n=354). One retrospective cohort study conducted in the US examined the cognitive function depending on the smoking status after HF diagnosis^24^. Both current (β=0.321; p=0.016; n=2043) and former smokers (β=0.204; p=0.041) had higher cognitive function than never smokers. One prospective cohort study conducted in the US examined the length of stay in the hospital, depending on the smoking status after HF diagnosis^22^. Smokers had similar length of stay in the hospital compared to non-smokers (relative risk, RR=0.37; 95% CI: 0.94–0.99; n=48612).

## DISCUSSION

Despite the strong association of smoking with cardiovascular disease and adverse events, little is known about the impact of persistent smoking after a HF diagnosis on patient outcomes. In this review, we found that persistent smoking after HF diagnosis, compared with non-smoking (former or never smoking), increased the risk of adverse health outcomes. In particular, our main finding was that current smokers, who continued smoking after HF diagnosis, were more susceptible to mortality and readmissions than non-smokers.

Our meta-analysis demonstrated that mortality was the most frequently examined variable out of the seven types of adverse health outcomes associated with smoking status. Furthermore, persistent smokers showed higher mortality than non-smokers. However, we could not verify whether the higher mortality among persistent smokers was primarily ascribable to the functional deterioration of the cardiovascular system or to other causes, given that most data used in the studies indicated ‘all-cause mortality’ as the cause of death. Specifically, chemical constituents of smoke have high oxidant and inflammatory power that can directly induce endothelial damage and potentiate inflammatory response^28-30^. Namely, cigarette smoking can promote atherosclerosis by its effects on lipid profile^25,31^. Moreover, according to one study^32^, nicotine along with carbon monoxide and oxidative stress induce fibrosis at different cardiac sites; thus, generating a structural remodeling that may favor cardiac arrhythmia. For these reasons, the damaged cardiovascular system induces myocardial systolic and diastolic dysfunction through smoking, thus aggravating HF symptoms^21^, which can result in death. Consequently, further studies are necessary to completely understand the exact mechanisms underlying the vascular damage induced by smoking. Such knowledge can help healthcare providers to educate active or heavy smokers after a HF diagnosis against smoking.

In previous studies, tobacco smoking was associated with higher risk of cognitive decline. These associations are thought to be caused mainly by smoking-related damage, which develops during cardiovascular and respiratory processes in the brain^33,34^. However, in a study in which cognitive function was reported depending on the smoking status, current smokers were found to have higher cognitive function than never smokers^24^. This may be explained by the fact that a cognitive function decline is associated not only with smoking but also with other factors, such as age, hemoglobin level, and body mass index^35,36^. These factors act as confounding variables, and there is a need to adjust for them. Moreover, only one of the studies analyzed in this systematic review examined the association between cognitive function and smoking status after HF diagnosis, and the validity of its findings will have to be verified by additional research. In one study that examined the length of stay in the hospital in relation to smoking status, persistent smokers and non-smokers showed similar lengths of stay^22^. This study noted that the sudden smoking cessation after hospitalization might have resulted in the improved health status. Although the average length of stay in the hospital reported in this study was 4 days, too short a period of smoking cessation to improve health. Taken together, HF patients who are smoking at diagnosis may benefit from counseling regarding the harms of continued smoking after a diagnosis of HF, as one way to improve quit rates. Secondhand smoke exposure is related to increased risk of CVD and ischemic heart disease^37,38^. Thus, smoke-free policies within hospitals can help persistent smokers to attempt to quit smoking.

In our review, of 70461 participants, 11252 (16%) from nine studies continued smoking after HF diagnosis. Similar percentages were found in previous studies for diseases associated with smoking: 14.1% of 1700 patients with CVD^13^; 17.6% of 5185 patients diagnosed with cancer^39^; and 17% of 893 patients with lung cancer^40^. Most of the patients diagnosed with HF undergo interventions designed to prevent the aggravation of HF. Such interventions include smoking cessation, low-salt diet, abstention from drinking, regular exercise, and management of risk factors such as hypertension, diabetes, and hyperlipidemia^41^. The finding of this study showing that 16% of patients with HF failed to quit smoking highlights the need of customized health education for the patients by identifying the causes of persistent smoking and negative attitudes towards health-promoting behaviors.

Our review of the studies included, found that the follow-up period (12–41 months) was not sufficiently long to verify the adverse health outcomes for persistent smoking after a HF diagnosis. A cohort study should be performed to clearly indicate the temporal sequence between exposure and outcome, or with a long follow-up period in case of a low incidence rate^42^. In particular, a lifestyle variable like smoking requires a study design providing sufficient follow-up for the target outcome, given its long-term negative health consequences. Furthermore, the smoking intensity, such as cumulative smoking in pack-years, number of cigarettes smoked per day, and duration of smoking, was not sufficiently examined. In previous studies, dose effect association between pack-years of exposure and HF risk was observed^43^. Furthermore, current smokers’ risk of HF increased as their daily tobacco consumption increased^44^. That is, since smoking affects the cardiovascular system to a greater or lesser extent depending on smoking intensity, strategies for smoking reduction will have to be developed by investigating the threshold smoking intensity for exacerbating HF. For HF diagnosis, medical records (objective data) were used in all studies, while NYHA functional class, LVEF, and BNP, were used to verify the severity of HF. NYHA functional class is a subjective measure useful for determining the motor skills and symptoms of patients with HF and widely used for monitoring patients with HF^45^. NYHA functional class and LVEF were well documented in 77.8% of the included studies, to describe the subjective and objective characteristics of the patients with HF. When the level of LVEF is ≥40%, the ventricle volume increases, and pressure overload must be monitored by additionally checking the increase in BNP^46,47^. Of the two studies reporting LVEF ≥40% in this systematic review, only one investigated BNP. To ensure a clear explication of the association between smoking status after HF diagnosis and adverse health outcomes, NYHA, BNP and LVEF will have to be clearly determined as the diagnostic criteria for HF.

### Strengths and limitations

Our systematic review has several strengths. We performed a comprehensive review of various databases to identify the health outcomes reported to be caused by persistent smoking after HF diagnosis. All papers were quality assessed using a robust method. Also, five of the seven adverse health outcomes – mortality, readmission, ventricular tachycardia, arterial stiffness, length of stay – were collected through medical records and can be regarded as objective information, which reduces the risk of bias. We provided a detailed description of HF-related patient characteristics by identifying the related objective (LVEF, BNP) and subjective (NYHA functional class) symptoms. Most of all, this study found evidence that suggests negative effects with persistent smoking, on the various health outcomes. There is a need for further studies to identify mechanisms or pathways between smoking and adverse health outcomes in HF patients. Awareness among healthcare providers of the adverse effects of smoking on the progression of HF should be increased.

This study has some limitations. In the present observational study, we found it difficult to control for relative confounding directly. We were also not able to investigate problems with natural confounding in the original studies. Most of the studies included did not categorize the type of HF, such as HF with preserved ejection fraction (HFpEF) and reduced ejection fraction (HFrEF). Previous studies showed that risk factors could be different between HFpEF and HFrEF^46,47^. Specifically, the smoker status has been associated with structural changes with increase in left ventricular mass compared with nonsmokers^10^. With regard to this phenomenon, this study was not able to explain in detail the harmful impact, between HFpEF and HFrEF. We did not provide sufficient information regarding the characteristics of patients with HF because only two studies reported the duration of HF. Since smoking status was self-reported or evaluated based on medical records, it might not have provided adequate objective measures compared with biochemical tests. Most of these studies were conducted in the US (77.8%), hence the results of the present study lack generalizability to other populations.

## CONCLUSIONS

Our study showed that approximately 16% of smokers continued smoking even after HF diagnosis. This supports the need to assess patients’ smoking status during outpatient follow-up visits. Importantly, our review identified that adverse health outcomes were more common in persistent smokers after HF diagnosis than in non-smoker patients. Healthcare professionals should create awareness regarding the harm of continued smoking in HF patients. Particularly, patients willing to try to reduce or stop smoking should be encouraged. Further studies are needed to explore underlying reasons why HF patients continue to smoke after a HF diagnosis, in order to develop patient-centered smoking cessation strategies. Furthermore, the causal relationships between persistent smoking and various aspects of adverse health outcomes can be determined with a large and culturally diverse HF population.
